# Supplementary material for: Psychometric development and evaluation of a COVID-19 social stigma scale in Indonesia
Source: PLoS One. 2023 Apr 5;18(4):e0283870. doi: 10.1371/journal.pone.0283870 (PMC10075483; doi:10.1371/journal.pone.0283870)
Supplement: S1 Appendix — (DOCX) [file pone.0283870.s001.docx]

**Appendix. Final Version The seven-dimensional COVID-19 Social Stigma Scale**

**Use the coding:**

**1 : Strongly disagree**

**2 : disagree**

**3 : unsure**

**4 : agree**

**5 : strongly agree**

| **No.** | **Item** | **Strongly disagree** | **Disagree** | **Unsure** | **Agree** | **Strongly agree** |
| --- | --- | --- | --- | --- | --- | --- |
|  | **Dimension 1: Social Distancing** |  |  |  |  |  |
| **1** | People close themselves off because they are afraid of being blamed by others. |  |  |  |  |  |
|  | **Dimension 2: Traditional Prejudice** |  |  |  |  |  |
| **2** | COVID-19 sufferers are afraid of their identity being shared with the public. |  |  |  |  |  |
| **3** | People feel ashamed when diagnosed with COVID-19. |  |  |  |  |  |
|  | **Dimension 3: Exclusionary Sentiments** |  |  |  |  |  |
| **4** | People do not want to report being exposed to COVID-19 because they are ashamed and afraid of being shunned. |  |  |  |  |  |
| **5** | People would not admit they are infected with COVID-19 because they are afraid of losing their jobs. |  |  |  |  |  |
|  | **Dimension 4: Negative Affect** |  |  |  |  |  |
| **6** | People still feel that suffering from COVID-19 is a disgrace. |  |  |  |  |  |
| **7** | People with symptoms of anosmia don’t want to do swab tests for fear of being disgraced. |  |  |  |  |  |
|  | **Dimension 5: Treatment Carryover** |  |  |  |  |  |
| **8** | People hide their status of being exposed to COVID-19 for fear of losing their jobs. |  |  |  |  |  |
|  | **Dimension 6: Disclosure Carryover** |  |  |  |  |  |
| **9** | People do not want to do a rapid test for fear of a positive result. |  |  |  |  |  |
| **10** | People prefer self-isolation without being tested, but they do not commit to doing self-isolation properly. |  |  |  |  |  |
|  | **Dimension 7: Perception of Dangerousness** |  |  |  |  |  |
| **11** | People do not want to mention their close contacts when tracing for fear of losing other people’s friendship. |  |  |  |  |  |
